# *Nigella sativa* Pretreatment in Guinea Pigs Exposed to Cigarette Smoke Modulates In Vitro Tracheal Responsiveness

**DOI:** 10.5812/ircmj.10421

**Published:** 2014-07-05

**Authors:** Rana Keyhanmanesh, Hossein Nazemiyeh, Hossein Mazouchian, Mohammad Mahdi Bagheri Asl, Mahdi Karimi Shoar, Mohammad Reza Alipour, Mohammad Hossein Boskabady

**Affiliations:** 1 Drug Applied Research Center, Tabriz University of Medical Sciences, Tabriz, IR Iran; 2 Department of Physiology, Tabriz University of Medical Sciences, Tabriz, IR Iran; 3 Tuberculosis and Lung Research Center, Tabriz University of Medical Sciences, Tabriz, IR Iran; 4 Pharmaceutical Nanotechnology Research Center, Tabriz University of Medical Sciences, Tabriz, IR Iran; 5 Student Research Committee, Student Research Center, Tabriz University of Medical Sciences, Tabriz, IR Iran; 6 Departmentof Physiology, Medical School and Pharmacological Research Centre of Medical Plants, Mashhad University of Medical Sciences, Mashhad, IR Iran

**Keywords:** *Nigella sativa*, Tobacco Products, Pulmonary Disease, Chronic Obstructive

## Abstract

**Background:**

In previous studies, the bronchodilator and antitussive effects of *Nigella sativa* have been demonstrated on guinea pigs.

**Objectives:**

In the present study, the effect of the hydroethanolic extract of *N*.* sativa* on tracheal responsiveness in guinea pigs exposed to cigarette smoke was examined.

**Materials and Methods:**

Three groups of guinea pig models of COPD were given drinking water alone (COPD group), drinking water containing vitamin C (COPD + VC group), and *N*.* sativa* (COPD + NS group). Tracheal responses to methacholine were measured as effective concentration causing 50% of maximum response (EC50 M) in control animals (group C) and three groups of guinea pigs with COPD (n = 7, for all groups). Tracheal responses to 0.1% ovalbumin in comparison to contraction obtained by 10 µM methacholine were also examined.

**Results:**

The tracheal responsiveness to both methacholine and ovalbumin in guinea pigs with COPD were significantly higher than those of controls (P < 0.001 for both cases). The tracheal responsiveness in the COPD + VC and the COPD + NS groups to both methacholine and ovalbumin were significantly decreased in comparison to the COPD group (P < 0.05 and P < 0.001, respectively).

**Conclusions:**

These results showed the preventive effect of hydroethanolic extract of *N*.* sativa* on tracheal responsiveness of guinea pig model of COPD, which was as effective as vitamin C.

## 1. Background

Chronic obstructive pulmonary disease (COPD) is one of the most prevalent lung diseases that makes the breathing difficult. There are two main forms of COPD: chronic bronchitis, which involves a long-term cough with mucus production; and emphysema that destructs the lungs parenchyma over time. Smoking is the leading cause of COPD. Other risk factors include exposure to certain gases or fumes in the workplace, exposure to tremendous amounts of second hand smoke and pollution, and frequent use of cooking fire without proper ventilation (1-3).

There is no cure for COPD. The best way to slow down the lung damage is to stop smoking. Some medications are used to treat COPD including bronchodilators, steroids, and anti-inflammatory drugs; although these drugs can relieve symptoms, many side effects and drug tolerance may develop. Hence, nowadays the physicians try to study the therapeutic effect of herbal medicines such as *Nigella sativa* (black seeds), an annual herbaceous plant belonging to the *Ranunculaceae* family commonly used as a natural remedy for various diseases in Middle Eastern folk medicine for over 2000 years (4-6). All chemical constituents of the plant were summarized in a review by Salem (7).

Recently, clinical and animal studies have shown that the extracts of the black seeds have many therapeutic effects due to its bronchodilatory, immunomodulatory, antibacterial, antihistaminic, and antioxidative characteristics (8-14). Moreover, a previous study showed that different extracts of *N*.* sativa* had antitussive effect on the guinea pig (15).

## 2. Objectives

This present experimental study aimed to examine the effect of hydroethanolic extract of *N*.* sativa* on tracheal responsiveness of guinea pigs exposed to cigarette smoke, i.e. guinea pig model of COPD, in comparison with vitamin C, an antioxidant agent.

## 3. Materials and Methods

### 3.1. Plant and Extract

In this study, *N*.* sativa* was collected from northeast of Iran and identified by botanists in the herbarium of Ferdowsi University of Mashhad with the specimen number 293-0303-1. After drying its seeds at room temperature in the absence of sunlight, the hydroethanolic extract was prepared using a maceration method; 1000 g of chopped *N*.* sativa* seeds were mixed with 900 mL of 50% ethanol for 72 hours at 40℃. This process was repeated three times. The solutions were dried by rotary evaporation at 50℃ (16, 17). According to our previous study (18), the 1.25 g/L solution was prepared by adding 0.9% saline.

### 3.2. Animals and Cigarette Smoke Exposure

Experimental male guinea pigs (weight, 525 ± 32.8 g) were exposed to cigarette smoke in an awake, restrained, and spontaneously breathing state in a smoking chamber, which was a modification of that described by Boskabady et al. (14). This Plexiglas box consists of two chambers: head chamber (31 cm × 13 cm × 9 cm) and body chamber (31 cm × 13 cm × 28 cm). Three animals were placed in this Plexiglas box. The cigarette smoke was delivered by means of two syringes only to the head chamber. Twenty milliliter puffs of cigarette smoke were drawn out of the cigarettes with syringes and then exhausted at a rate of two puffs per minute into the animals’ head chamber. Exposure of animals to each cigarette lasted eight to nine minutes, with a ten-minute resting period between cigarettes. The animals were exposed initially to one commercial nonfiltered cigarette per day, which was gradually increased to a maximum of five cigarettes per day over a two-week period. The exposure to the smoke of five cigarettes per day, six days per week, continued for three months.

The study was approved by the Ethic Committee of the Tabriz University of Medical Sciences. The control animals (group C) were placed in the Plexiglas box but were exposed to normal saline. The experimental animals, kept in an animal house in controlled temperature room (22℃-24℃) with humidity of 40% to 60% and light period (12 hours light-12 hours dark cycle). Food and water were available ad libitum. They were given various types of drinking water during this protocol as follows (n = 7 for each group):

1. drinking water alone (group C);
2. drinking water alone (group COPD, an animal model of COPD);
3. drinking water containing 0.25 g/L Vitamin C (Chemifarma pharmaceutical veterinary industry, Italy) (COPD + VC group); and
4. drinking water containing 1.25 g/L *N*.* sativa* extract (COPD + NS group).

### 3.3. Tissue Preparations

After protocol of induction, guinea pigs were sacrificed by a blow on the neck, and trachea was removed. Each trachea was cut into ten rings, each containing two to three cartilaginous rings. All the rings were sutured together to form a tracheal chain. Then the rings, except the terminal ring, were cut open opposite the trachealis muscle to clarify the muscular response (19). Finally, tissue was suspended in a 20-mL organ bath (Schuler organ bath type 809, March-Hugstetten, Germany) containing Krebs-Henseliet solution with the following composition: NaCl, 120 mM; NaHCO_3_; 25 mM; MgSO_4_, 0.5 mM; KH_2_PO_4_, 1.2 mM; KCl, 4.72 mM; CaCl_2_, 2.5 mM; and dextrose, 11 mM. The Krebs solution was maintained at 37℃ and was gassed by 95% O_2_ and 5% CO_2_. Tissue was suspended under isotonic tension of 1 g and allowed to equilibrate for at least one hour while it was washed with Krebs solution every 15 minutes.

Responses were detected using Vernier control sensor (type 850 N) with sensitivity range of zero to 20 g and resolution of 0.2 mm per turn (Hugo-Sachs Elektronik, Germany), amplified with an amplifier (ML/118 quadribridge amp, March-Hugstetten, Germany), and recorded on Powerlab recorder (ML-750, 4 channel recorder, March-Hugstetten, Germany).

### 3.4. Assessment of Tracheal Response to Methacholine

In each experiment, a cumulative log concentration-response curves of methacholine hydrochloride-induced contraction of tracheal chain were obtained. Consecutive concentrations (10^-7^ to 10^-2^ mM) of methacholine (Sigma Chemical Ltd, UK) were added every three minutes and the contraction due to each concentration was recorded at the end of each three minutes; the effect reached a plateau in all experiments. To obtain the curve, the percentage of contraction due to each methacholine concentration in proportion to the maximum contraction obtained by its final concentration was plotted against log concentration of methacholine.

The effective concentration of methacholine causing 50% of maximum response (EC_50_) was measured using methacholine response curve in each experiment. Contractility response to 10 μM methacholine as the magnitude of contraction was also measured.

### 3.5. Measurement of Tracheal Response to Ovalbumin

The tracheal response of all animals to 0.1% ovalbumin solution was measured by adding 0.5 mL of 4% ovalbumin solution to 20-mL organ bath, the degree of tracheal chain contraction after 15 minutes was recorded, and then the value were expressed as proportion (in percentage) to contraction obtained by 10 µM methacholine.

### 3.6. Statistical Analysis

One sample Kolmogorov-Smirnov test revealed that the data were normally distributed. The data of tracheal response to methacholine (EC_50_), tracheal contractility response, and tracheal response to ovalbumin were expressed as mean ± SEM. The data of COPD group were compared with control and treated guinea pigs using ANOVA. For statistically significant comparisons, post hoc analyses were performed using Tukey tests. The data were also compared between two groups of treated animals using independent samples t test. All these comparisons were done by means of InStat statistical software (Graphpad Software, San Diego, CA, USA). The level of significance was accepted at P < 0.05.

## 4. Results

### 4.1. Tracheal Response to Methacholine

**Figure 1.**
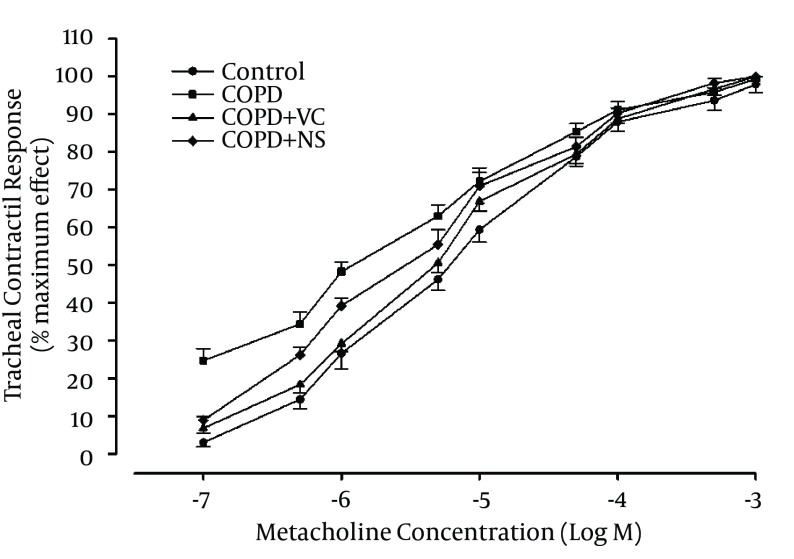
Cumulative Log Concentration-Response Curves of Methacholine-Induced Contraction of the Isolated Trachea in the Study Groups Abbreviations: COPD, chronic obstructive pulmonary disease; C, control group; COPD + VC, an animal model of COPD treated with vitamin C; and COPD + NS, an animal model of COPD treated with *Nigella sativa* (for each group, n = 7).

Concentration response curves to methacholine showed leftward shift of the curve in COPD group in comparison with the group C. On the other hand, the curves of COPD + VC and COPD + NS groups were shifted to right in comparison with the group COPD while they had leftward shift in comparison with the group C (Figure 1).

**Figure 2.**
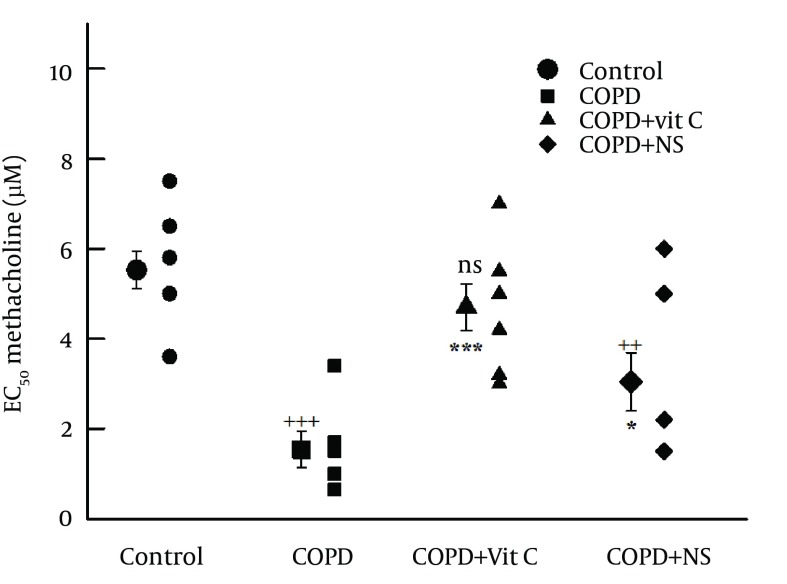
Individual Values and Means (Big Symbols With Bars) of Tracheal Response to Methacholine in the Study Groups ^a, b^ a) Abbreviations: COPD, chronic obstructive pulmonary disease; C, control group; COPD + VC, an animal model of COPD treated with vitamin C; and COPD + NS, an animal model of COPD treated with *Nigella sativa* (for each group, n = 7). b) Statistical differences between control and different groups: NS, no significant difference; +, P < 0.05; ++, P < 0.01; +++, and P < 0.001. Statistical differences between COPD + VC and COPD + NS vs. COPD group: NS, no significant difference;*, P < 0.05; **, P < 0.01; and ***, P < 0.001.

The mean value of EC_50_ in tracheal chains of the COPD group was significantly lower than group C (1.54 ± 0.40 and 5.25 ± 0.41 μM, respectively; P < 0.001) (Figure 2A). The mean value of EC_50_ in tracheal chains of pretreated groups, namely COPD + VC and COPD + NS groups (4.70 ± 0.52 and 3.04 ± 0.65 μM, respectively) were significantly improved in comparison with the COPD group (P < 0.001 and P < 0.05, respectively) (Figure 2A); however, the mean value of EC_50_ of tracheal chains of pretreated group with *N*.* sativa* were still significantly lower than that of group C (P < 0.01; Figure 2A). Mean value of EC_50_ in tracheal chains of COPD + NS group was insignificantly lower than that of COPD + VC group (Table 1).

**Table 1.** Values of Tracheal Response to Methacholine and Ovalbumin and Contractility in Study Groups ^a^

Parameters	C	COPD	COPD + VC	COPD + NS
**EC_50_, µmol**	5.25 ± 0.41	1.54 ± 0.40	4.70 ± 0.52	3.04 ± 0.65 (P = 0.070)
**OA, %**	9.25 ± 2.13	40.62 ± 3.95	26.5 ± 2.76	13.66 ± 3.88
**Contractility**	1.35 ± 0.09	1.77 ± 0.12	1.37 ± 0.06	1.28 ± 0.10 (P = 0.477)

^a^ Abbreviations: EC50, values of tracheal response to methacholine; OA, ovalbumin; COPD, chronic obstructive pulmonary disease; C, control group; COPD + VC, an animal model of COPD treated with vitamin C; and COPD + NS, an animal model of COPD treated with Nigella sativa (for each group, n = 7).

### 4.2. Tracheal Response to Ovalbumin

Tracheal response to ovalbumin in chains of COPD group (40.62% ± 3.95%; range, 20%-55%) was significantly higher than in group C (9.25% ± 2.13%; range, 0-15%; and P < 0.001) (Figure 2B). Tracheal response to ovalbumin in pretreated groups, namely, COPD + VC (26.5% ± 2.76%; range, 12%-35%) and COPD + NS (13.66% ± 3.88%; range, 0-30%) groups, was significantly improved in comparison with group C (P < 0.001 for both) (Figure 2B); however, tracheal response to ovalbumin was still significantly greater in COPD + NS group than in group C (P < 0.001) (Figure 2B). Tracheal response to ovalbumin was significantly lower in COPD + NS than in COPD + VC groups (P < 0.05, Table 1).

### 4.3. Contractility

**Figure 3.**
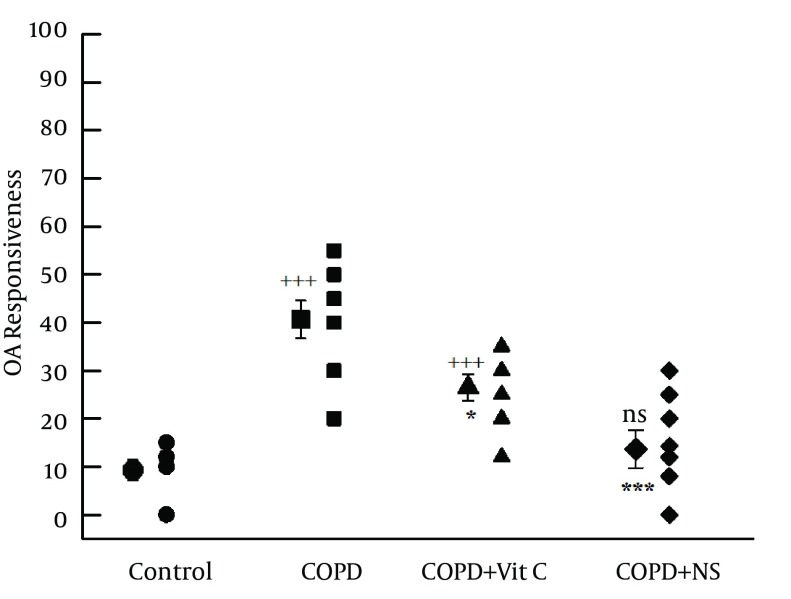
Individual Values and Mean (Big Symbols With Bars) of Tracheal Response to Ovalbumin (Percent Concentration in Proportion to Contraction Obtained by 10 µM Methacholine) ^a, b^ a)Abbreviations: COPD, chronic obstructive pulmonary disease; C, control group; COPD + VC, an animal model of COPD treated with vitamin C; and COPD + NS, an animal model of COPD treated with Nigella sativa (for each group, n = 7). b) Statistical differences between control and different groups: NS, no significant difference; +, P < 0.05; ++, P < 0.01; +++, and P < 0.001. Statistical differences between COPD + VC and COPD + NS vs. COPD group: NS, no significant difference;*, P < 0.05; **, P < 0.01; and ***, P < 0.001.

**Figure 4.**
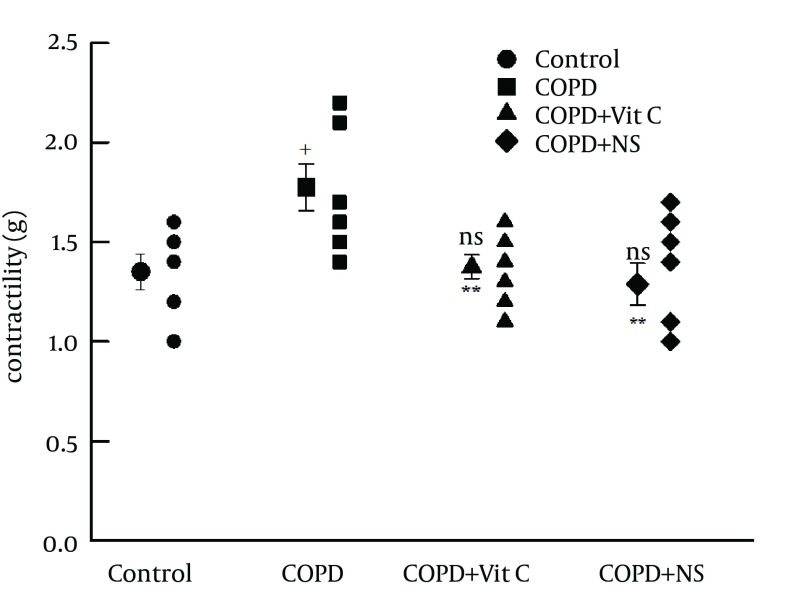
Tracheal Contractility Response to 10 μM Methacholine in the Study Groups ^a, b^ a) Abbreviations: COPD, chronic obstructive pulmonary disease; C, control group; COPD + VC, an animal model of COPD treated with vitamin C; and COPD + NS, an animal model of COPD treated with Nigella sativa (for each group, n = 7). b) Statistical differences between control and different groups: NS, no significant difference; +, P < 0.05; ++, P < 0.01; +++, and P < 0.001. Statistical differences between COPD + VC and COPD + NS vs. COPD group: NS, no significant difference;*, P < 0.05; **, P < 0.01; and ***, P < 0.001.

The contractility response of tracheal chains to methacholine of COPD group was significantly higher than that of group C (P < 0.05). The contractility response in pretreated groups were significantly lower in comparison with the COPD group (P < 0.01). There was no significant difference in the contractility response between any of the pretreated groups and group C (Figure 3). There was not any significant difference between the contractility response in COPD + VC and COPD + NS groups (Figure 4).

## 5. Discussion

In the present study, the preventive effect of long-term administration of the hydroethanolic extract of *N*.* sativa* on tracheal responsiveness to methacholine and ovalbumin were examined in guinea pigs exposed to cigarette smoke (guinea pig model of COPD). The results showed increased contractility response and tracheal responsiveness to methacholine and ovalbumin in guinea pigs with COPD in comparison with the controls. Pretreatment of these animals with Vitamin C and *N*.* sativa* prevented the increased tracheal responsiveness to methacholine and ovalbumin as well as the increased contractility response.

It is not fully understood how tobacco smoke and other inhaled particles damage the lungs and cause COPD. The common feature of COPD is the development of an inflammatory response characterized by activation of epithelial cells and resident macrophages and the recruitment and activation of neutrophils, eosinophils, monocytes, and lymphocytes. The activation of these cells generates O_2_, which is rapidly converted to H_2_O_2_ by superoxide dismutase (20).

The most important processes causing lung damage are oxidative stress produced by the high concentrations of free radicals in tobacco smoke and cytokine release due to inflammatory response to irritant particles such as tobacco smoke in the airways (21). Smoking and exacerbations of COPD result in decreased antioxidant capacity in plasma that can be explained by the increased radical oxygen species release from peripheral blood neutrophils (22, 23) or decreased levels of major plasma antioxidants such as vitamin C or E in smokers (18, 24). The decrease in antioxidant capacity in smokers occurs transiently during smoking and resolves rapidly after smoking cessation (22).

All prophylactic drugs used in treatment of COPDs should aim to reduce airway inflammation to reverse airway narrowing and limited effectiveness of the lungs. Therefore, the preventive effect of long-term administration of *N*.* sativa* on tracheal responsiveness to methacholine and ovalbumin might be due to its suppressing effect on airway inflammation. In fact, the inhibitory effects of the essential oil of *N*.* sativa* have been shown on both cyclooxygenase and 5-lipooxygenase pathways of arachidonic acid metabolism as well as on the membrane lipid peroxidation (8, 25).

In this study, one concentration of the hydroethanolic extract of *N*.* sativa* was administered during the protocol. This dose was obtained from our previous study (16). In that investigation, the effects of two doses of this extract were studied on tracheal chains of asthmatic guinea pigs and the results showed that there were no significant differences between their effects.

In this study, vitamin C was administered as positive control. Vitamin C or L-ascorbic acid, is an essential nutrient for humans and certain animal species. Vitamin C is a cofactor in at least eight enzymatic reactions. Ascorbic acid is well known for its antioxidant activity by acting as a reducing agent to reverse oxidation in liquids; hence, it has an effect on some diseases such as chronic inflammatory diseases (26-29).

Previous study (30) demonstrated that administration of vitamin C could markedly decrease the severity of inflammatory diseases such as COPD and elevation of vitamin C in serum would be a good prognostic indicator for diseases evaluation. In present study the pretreatment of animals, which were exposed to cigarette smoke (guinea pig model of COPD), with *N*.* sativa* was as effective as administration of Vitamin C on tracheal responsiveness. Therefore, as indicated in ancient Iranian medical books, *N*.* sativa* could have therapeutic effects on respiratory diseases including COPD.

In conclusion, the results of the present study indicated a preventive effect of *N*.* sativa* on tracheal responsiveness to methacholine and to a less extent to ovalbumin in guinea pigs with COPD.
